# Broad Th2 neutralization and anti‐inflammatory action of pentosan polysulfate sodium in experimental allergic rhinitis

**DOI:** 10.1002/iid3.164

**Published:** 2017-05-12

**Authors:** Caroline Sanden, Michiko Mori, Prajakta Jogdand, Jimmie Jönsson, Ravi Krishnan, Xiangdong Wang, Jonas S. Erjefält

**Affiliations:** ^1^ Unit of Airway Inflammation Department of Experimental Medical Science Lund University Lund Sweden; ^2^ Medetect AB Lund Sweden; ^3^ Paradigm Biopharmaceuticals Ltd. Melbourne, Victoria Australia; ^4^ Zhongshan Hospital Institute of Clinical Science Shanghai Institute of Clinical Bioinformatics Shanghai China

**Keywords:** Allergy, pentosan polyphosphate sodium, Th2 cytokines, interleukin‐4, interleukin‐5, interleukin‐13

## Abstract

**Background:**

Th2 cytokines like interleukin‐4, ‐5, and ‐13 are regarded as important drivers of the immunopathology underlying allergic rhinitis (AR) and asthma. The present study explores the capacity of pentosan polysulfate sodium (PPS), a semi‐synthetic heparin‐like macromolecular carbohydrate, to bind Th2 cytokines and exert biological neutralization in vitro, as well as anti‐inflammatory actions in vivo.

**Methodology:**

The capacity of PPS to bind recombinant Th2 cytokines was tested with surface plasmon resonance (SPR) technology and biological Th2 neutralization was assessed by Th2‐dependent proliferation assays. The in vivo anti‐inflammatory action of PPS was studied using a validated Guinea‐pig model of AR.

**Results:**

Binding studies revealed a strong and specific binding of PPS to IL‐4, IL‐5, and IL‐13 with IC values suggesting as stronger cytokine binding than for heparin. Cytokine binding translated to a biological neutralization as PPS dose dependently inhibited Th2‐dependent cell proliferation. Topical administration of PPS 30 min prior to nasal allergen challenge of sensitized animals significantly reduced late phase plasma extravasation, luminal influx of eosinophils, neutrophils, and total lavage leukocytes. Similar, albeit not statistically secured, effects were found for tissue leukocytes and mucus hyper‐secretion. The anti‐inflammatory effects of PPS compared favorably with established topical nasal steroid treatment.

**Conclusion:**

This study points out PPS as a potent Th2 cytokine‐binding molecule with biological neutralization capacity and broad anti‐inflammatory effects in vivo. As such PPS fulfills the role as a potential candidate molecule for the treatment of AR and further studies of clinical efficacy seems highly warranted.

## Introduction

Allergic rhinitis (AR) is an inflammatory disease of the upper airways with a global effect on a large subpopulation of individuals and impacting profoundly on the health and occupational productivity 1, 2.

The development of an allergic inflammation is orchestrated by a variety of cell mediators. Among the key drivers are Th2 cytokines like IL‐4, IL‐5, IL‐9, and IL‐13 3, 4. For example, IL‐4 and IL‐13 are instrumental for the differentiation of B cells and IgE isotype switching 5. IL‐5 produced locally at sites of allergic inflammation acts remotely on the bone marrow to disseminate eosinophils that in turn are regarded as important effector cells in AR due to their release of cytotoxic granule proteins 3, 6. Notably, apart from the classical production of Th2 cytokines by Th2 CD4^+^ T‐helper lymphocytes, the more newly discovered ILC2 innate lymphoid cells have recently emerged as a potentially important Th2 cytokine source in AR 7, 8.

The allergic reaction is classically divided into two major phases. In the early acute phase allergen‐IgE binding and cross‐linking of surface‐bound IgE on mast cells evokes a rapid degranulation, release of mediators such as histamine, and the manifestation of an acute phase allergic reaction 6, 9. The late‐phase is characterized by release of chemokines, Th2 cytokines, and recruitment of effector cells like eosinophils. Hence, while the early‐phase response to allergen exposure produces acute symptoms, the late‐phase response sustains and aggravates the local tissue inflammation.

Due to their proposed key role in allergic inflammation, Th2 cytokines have been in focus for novel treatment strategies. A plethora of neutralizing antibodies (biologics) targeting Th2 cytokines is currently in various phases of clinical development. Although some clinical efficacy has been demonstrated, this class of drugs has so far mainly been trialed in severe asthma 10, 11. Potential drawbacks with biologics directed against specific cytokines or their receptors are high treatment costs and the risk of limited efficacy due to mono‐specificity 11. Multiple cytokine‐targeted therapy has been attempted with GATA3‐specific DNA enzyme (DNAzyme) which binds and cleaves GATA3 mRNA resulting in the coordinated inhibition of Th2 cytokine transcription 12. GATA3‐specific DNAzyme administered by inhalation significantly attenuated both the early‐ and late‐phase asthmatic responses 13. However, this approach has yet to be trialed in AR.

The introduction of second‐generation, intranasal corticosteroids (INCS) for AR treatment, including beclomethasone, budesonide, mometasone, and fluticasone has evidently demonstrated reduced systemic effects with long‐term usage 14, 15. Nevertheless, patient dissatisfaction has been identified in AR surveys urging the need for new treatment options 2, 16. In light of the redundancy regarding the effector molecules involved in an allergic inflammation, the introduction of steroid‐independent but still broad neutralization of allergy‐associated cytokines and chemokines seems like an attractive treatment strategy.

Pentosan polysulfate sodium (PPS) is a semi‐synthetic heparin‐like macromolecular carbohydrate derivative that resembles glycosaminoglycans. PPS is manufactured from beechwood hemicellulose by sulfate esterification and has a molecular weight range of 4–6 kDa 17. PPS has been used in the clinic for its anti‐inflammatory and regenerative properties in the treatment of interstitial cystitis 18. In non‐allergic conditions, the anti‐inflammatory effects of PPS have been suggested to in part be mediated by inhibition NF‐kappa‐β activation 19, 20. Of relevance to AR, PPS is a potent inhibitor of histamine release from mast cells demonstrating fast action and sustained response compared to cromolyn 21. An additional important aspect of PPS is the structural similarities to heparin. Heparin has well established Th2 cytokine binding properties 22, 23, a feature that is most likely also valid for PPS. Heparin has been investigated in allergic respiratory models but due to its anti‐coagulant property, it has not been deemed attractive for clinical development 24.

With low anti‐coagulant activity and biochemical properties of PPS that suggest a multiple action on key allergic events, the potential use of PPS as an anti‐allergic treatment merits further investigation. In particular, the capacity of PPS to bind and neutralize key Th2 cytokines needs to be explored. Furthermore, the anti‐inflammatory actions of PPS under relevant in vivo conditions also remain to be studied.

The present study demonstrates a significant binding activity of PPS to Th2 cytokines and shows that this binding translates into biological inhibition of Th2 cytokine‐dependent cell responses. Furthermore, the in vivo anti‐allergic efficacy of intranasal PPS administration was tested using an established guinea‐pig AR model with known translational predictability. Our data demonstrate significant in vivo anti‐inflammatory actions of PPS that compares favorably to a head‐to‐head comparison with standard nasal steroid treatment.

## Materials and Methods

### Analysis of PPS binding to Th2 cytokines

The binding of recombinant human Th2 cytokines (IL‐4, IL‐5, and IL‐13) to PPS (Bene Pharma Chem, Geretsried, Germany) was analyzed with surface plasmon resonance (SPR) 25, 26. Immobilized heparin (a well‐known binder of Th2 cytokines) was used as reference binding molecule as previously described 25, 26. Biotinylated heparin was immobilized on CM‐4 sensor chips pre‐coated with streptavidin, and real‐time biomolecular interaction analyses were performed with a BIAcore 2000 SPR biosensor 25, 26. The affinity of the binding of IL‐4, IL‐5, and IL‐13 to PPS was determined by estimating the amount of PPS, required to inhibit cytokine binding to the heparin coupled biosensor surface (soluble exogenous heparin was used as cytokine‐binding reference molecule).

### Th2 cytokine dependent cell proliferation assays

Th2‐responsive cell lines were used to assess the capacity of PPS to antagonize IL‐4, IL‐5, or IL‐13 dependent proliferation in vitro. TF‐1.8, a subclone of the erythroleukemic cell‐line TF‐1 27, was used to assess the antagonistic effect of PPS on IL‐4 and IL‐13‐induced proliferation. The proliferation of the TF‐1.8 cell line is responsive to IL‐4 and IL‐13 and has been transfected to express the luciferase reporter gene for proliferation studies 28. The antagonistic effect of PPS against IL‐5 was explored with Ba/F‐IL‐5 cells. This IL‐5 responsive cell line is derived from the Ba/F3 cell line that was stably transfected with the human IL‐5 receptor α‐chain and the luciferase reporter gene as described by Coombe et al. 28. In these proliferation assays, proliferation is induced by the cytokine of interest and the level of luciferase activity, which directly reflects the cell numbers 29, was measured in the presence of different concentrations of antagonizing PPS or heparin.

### In vivo effects of topical PPS on nasal allergic inflammation

**Figure 1:**
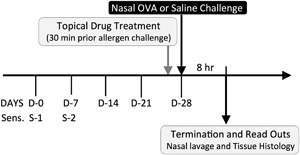
Schematic overview of the in vivo study design. Guinea pigs were sensitized to OVA at day 0 (sensitization 1) and day 7 (sensitization 2). Three weeks after the last sensitization, at day 28, animal groups were pre‐treated with either Budesonide (*n* = 10) or PPS (0.5 mg/kg, *n* = 10 or 5 mg/kg, *n* = 10) for 30 min prior to OVA or Saline challenge (control groups received PBS pre‐treatment, (*n* = 7 per control group). Changes in the late phase response were determined by analysis of nasal lavage fluid and histological nasal tissue (septum) at 8 h after allergen challenge.

The anti‐inflammatory actions of PPS were explored in a validated drug screening guinea‐pig model of AR 30. The in vivo study design is presented in Figure 1. Guinea pigs, Dunkin–Hartley males (total *n* = 60), 350 g, from Mollegaard Breeding Center, Denmark) were sensitized to ovalbumin (OVA) twice (on days 0 and 7) by an intraperitoneal injection of 0.5 mL saline containing l00 mg Al(OH)3 and 2 µg ovalbumin (OVA, Sigma–Aldrich, St. Louis, MO, US) 30, 31. The animals were housed in a modern, temperature and humidity‐controlled animal facility and with water and food ad libitum. Three weeks after the last sensitization, animals were anesthetized and the nasal cavity was exposed to allergen by dropping OVA solution at 20 mg/mL into bilateral nasal cavities. For the negative control, the animals received sensitization and were challenged with saline. Animals were randomized into treatment groups, sedated (1 mL/kg of Xylazine and Ketamine, 2:3 ration) and pretreated with either vehicle or drug (0.5 or 5 mg/kg of PPS, Bene Pharma or clinical formulation of Budesonide, Rhinocort Aqua, AstraZeneca, total dose of 0.65 mg steroid/kg), administered in a volume of 25 μL per nostril 30 min prior to intranasal instillation of OVA. Animals were terminated by an overdose of pentobarbital sodium (Mebumal) 8 h after the allergen provocation and samples were collected for analysis of read out parameters. All animal experiments have been approved by the local Malmo/Lund animal ethical committee in Sweden (DNo:M7907).

#### Analysis of nasal lavage fluid (NLF)

The NLF was collected by gently rinsing the nasal cavities with phosphate‐buffered saline 30. The cells in the NLF were centrifuged and resuspended in PBS and counted using a semi‐automated hematology analyzer. Allergen‐induced late phase extravasation of protein‐rich and non‐sieved plasma into the nasal cavities was measured as total lavage supernatant protein content using BioRad protein assay. The cell composition of the nasal lavage was quantified after a cytospin preparation (Shandon, UK) and leukocyte differential staining with May–Grunwald–Giemsa.

#### Histopathological analysis of nasal tissue

From each animal the nasal septum was carefully dissected out, subjected to overnight fixation in 4% buffered formaldehyde, dehydrated, and embedded in paraffin. From each septum, series of 3 μm sections (transverse section plan) were obtained from two depth levels, separated by 200 μm. Sets of sections from both depth levels were subjected to standard hematoxylin staining, Periodic Acid‐Schiff (PAS) staining (for goblet cell evaluation), FITC eosinophil granule staining 6, 32, and immunohistochemical (IHC) staining of T lymphocytes. Sections for IHC were subject to Heat‐Induced Epitope Retrieval (HIER, PT‐link machine, Dako Cytomation, Denmark) prior to automated IHC staining (Dako Autostainer Robot, Dako) using a mouse anti‐guinea pig T cell monoclonal antibody (MCA751, clone CT5, AbD Serotec). For quantification, stained sections were digitalized in an Olympus VS‐120 slide scanner. The epithelial area was outlined my manual cursor tracing the histochemical staining for eosinophils, T cells, and PAS were automatically quantified by computerized image analysis (Visiomorph^DP^, Visiopharm, Denmark).

### Statistical analysis

Data were analyzed in GraphPad Prism v 6 (GraphPad Software, Inc., San Diego, CA). One‐way Anova followed by Dunnett's multiple comparison was used to test differences between OVA‐treated animals and SAL, BUD, or PPS study groups. Pearson parametric method was used to test correlations between levels of eosinophils, T lymphocytes, and mucus content. Data were presented as means and standard error of the mean, *P* values <0.05 were considered to be significant.

## Results

### PPS has strong binding affinity to IL‐4, IL‐5, and IL‐13 relative to heparin

**Figure 2:**
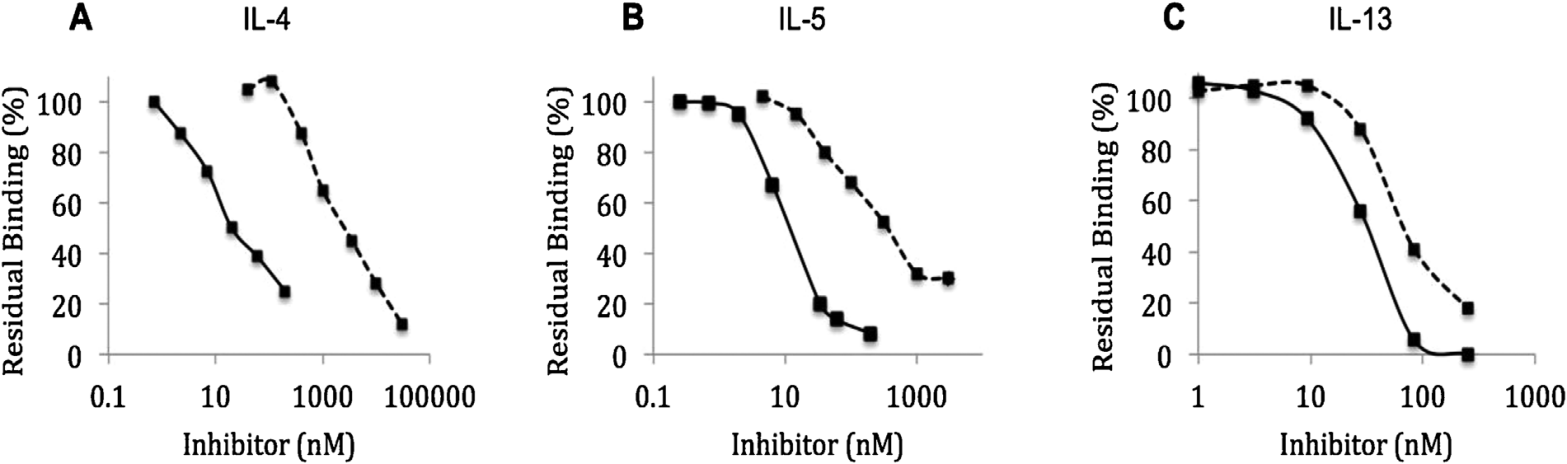
Molecular interactions and binding of PPS and heparin to Th2 cytokines as revealed by surface plasmon resonance (SPR) methodology. Data are shown for representative titration experiments showing the capacity of soluble heparin (dashed lines) and PPS (solid lines) to bind (A) IL‐4, (B) IL‐5, and (C) IL‐13 and prevent attachment to a cytokine‐binding detector chip. The experiments were performed on the BIAcore 2000 platform and with a heparinised CM4 flow cell detector 29.

Recombinant human (rh) rhIL‐4, rhIL‐5, rhIL‐13 readily bound to immobilized heparin (which is a well‐known Th2 cytokine binder). The binding was specific, as there was little interaction with sensor chips lacking heparin and the binding was dose‐dependently inhibited by exogenous heparin (Fig. 2A–C). Titration experiments revealed that compared to heparin PPS displayed a similar but stronger binding affinity to IL‐4, IL5, and IL‐13 (Fig. 2). The IC50 value for inhibition of IL‐4 binding was 25–80 nM for PPS and 3300 nM for exogenous heparin. Similar differences in inhibition potency were found for IL‐5 (IC50 for PPS 4–10 and 30–100 nM for heparin) and IL‐13 (IC50: PPS 28 vs. 90 nM for free heparin).

### Antagonistic effects of PPS on IL‐4, IL‐5, and IL‐13‐dependent cell proliferation

**Figure 3:**
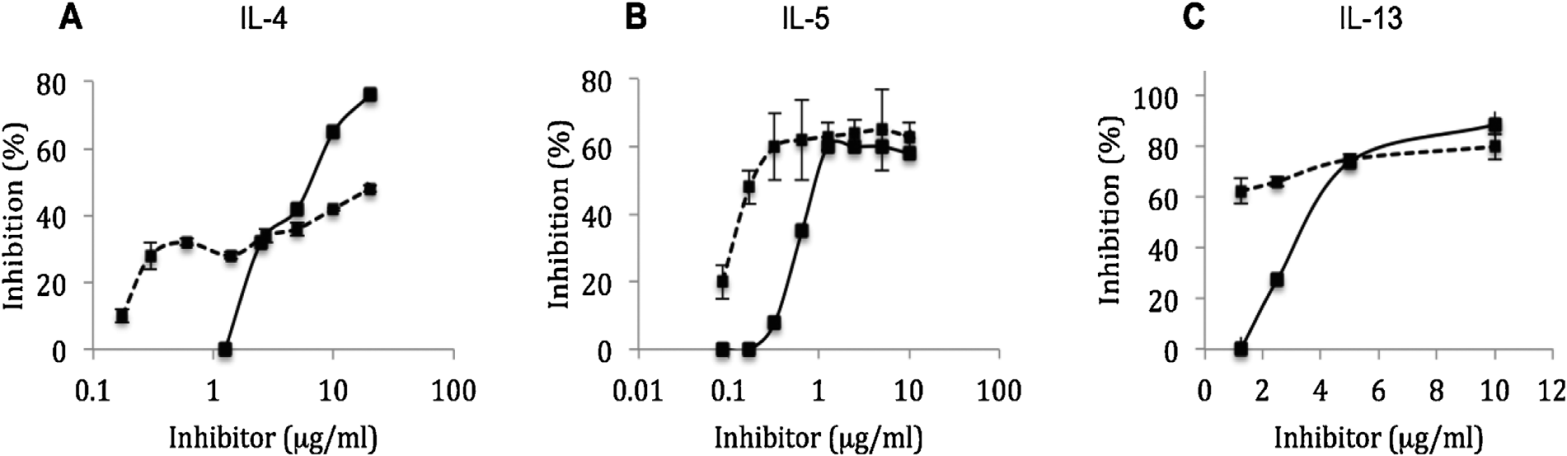
Dose–response curves of the capacity of PPS (solid line) and heparin (dashed line) to inhibit cytokine‐dependent cell proliferation in vitro. Data are shown for representative experiments. The cell densities were measured as luciferase activity 28 by a Victor 1420 Multi‐label counter (Wallac, Turku, Finland) after 48 h incubation in RPMI/5% 20 w/v FCS in the presence of 2.5 ng/mL stimulating cytokine: recombinant (A) IL‐4, (B) IL‐5, and (C) IL‐13.

A dose–response inhibitory effect of PPS and heparin on the IL‐4 dependent proliferation of TF‐1.8 cells was demonstrated in the range of 1.25–50 μg/mL in the presence of 2.5 ng/mL IL‐4 (Fig. 3A). PPS showed a 30% inhibition of TF‐1.8 cell proliferation at 2.5 μg/mL and a maximal inhibition of 78%, compared to 45% by heparin at 50 μg/mL.

PPS also inhibited IL‐5‐dependent proliferation of the IL‐5 responsive cell line Ba/F‐IL‐5 in a dose‐dependent manner in the presence of 2.5 ng/mL of IL‐5. A 10% inhibition of proliferation of the Ba/F‐IL‐5 cells was noted at a low dose of 0.5 μg/mL of PPS and a inhibition of 60% occurred at a concentration of 1.25 μg/mL (Fig. 3B). PPS showed a parallel IL‐5 antagonistic activity to heparin.

The dose–response curve for the inhibition of IL‐13 dependent proliferation of TF‐1.8 cells in the presence of 2.5 ng/mL of IL‐13 is shown in Figure 3C. Heparin demonstrated a 60% inhibition of TF‐1.8 cell proliferation at a low dose of 1.25 μg/mL compared to no effect by PPS at the same dose. However both agents showed a peak inhibition of about 80% at 10 μg/mL.

### In vivo efficacy of PPS on allergen‐induced plasma extravasation and immune cell recruitment in sensitized and allergen‐challenged guinea‐pigs

#### PPS reduces allergen‐induced leukocyte entry into the nasal cavity

**Figure 4:**
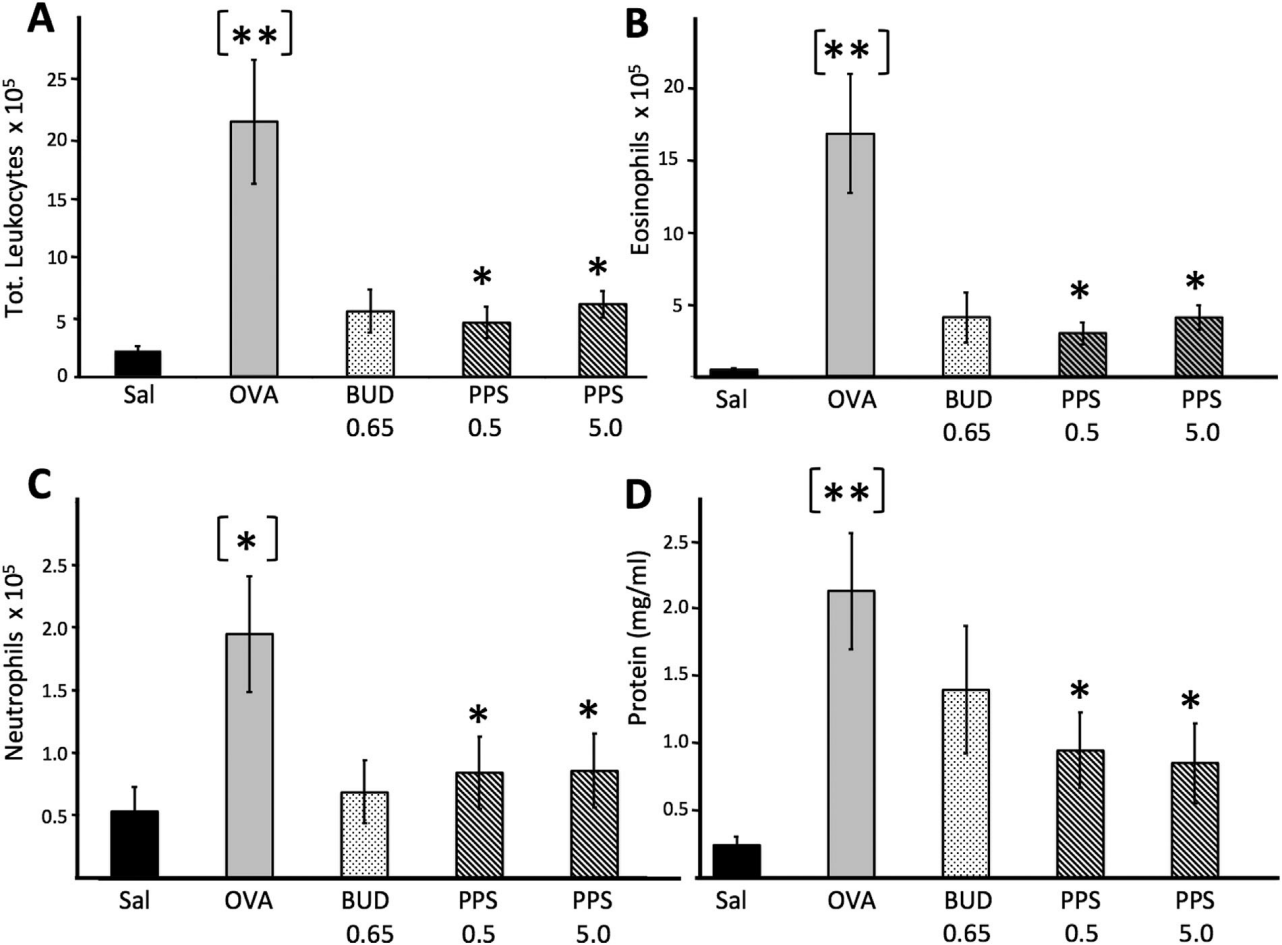
Capacity of PPS and budesonide to reduce allergen‐induced influx of nasal lumen leukocytes (A–C) and plasma extravasation (D). Samples for leukocytes and plasma exudation were collected during the allergic late phase (8 h). Data are expressed as cells/mL nasal lavage fluid. Plasma extravasation was expressed as total protein levels in nasal fluid supernatants 30. Asterisks in brackets denote statistical difference between saline and OVA exposed positive controls whereas asterisks denote difference between sham versus drug‐treated OVA‐exposed animals. PPS‐0.5 and 5 correspond to a prophylactic topical dose of 0.5 and 5 mg/kg, respectively.

Total leukocyte, eosinophil, and neutrophil numbers were significantly increased in NLF harvested from animals sensitized and challenged with OVA and sham treated with PBS, as compared to those challenged with PBS (*P* = 0.006, 0.004, and 0.023 in Fig. 4, respectively). Intranasal budesonide inhibited about 80% of OVA‐induced influx of leukocytes, eosinophils, and neutrophils in the NLF. Similar significant leukocyte reduction was shown for PPS where both 0.5 and 5 mg/kg resulted in about 80% inhibition on OVA‐induced influx of total leukocytes, eosinophils, and neutrophils (Fig. 4). The inhibition mediated by PPS was at both doses statistically significant on OVA‐induced influx of total leukocytes and eosinophils (Fig. 4A–C).

#### Reduced allergen‐induced plasma extravasation after topical PPS treatment

Plasma extravasation, measured as NLF total protein content 30, was significantly elevated in sensitized and OVA‐challenged animals compared to sensitized but sham‐treated controls (*P* < 0.01, Fig. 4D). Budesonide‐treated animals displayed reduced, albeit not statistically secured protein values compared to sham‐treated OVA‐challenged animals. Both at 0.5 and 5 mg/kg PPS‐treated animals displayed a significant (*P* < 0.05) and roughly 60% inhibitory effects on OVA‐induced increase of total protein content (Fig. 4D).

#### Effect of PPS on the nasal tissue parameters in allergic animals

**Figure 5:**
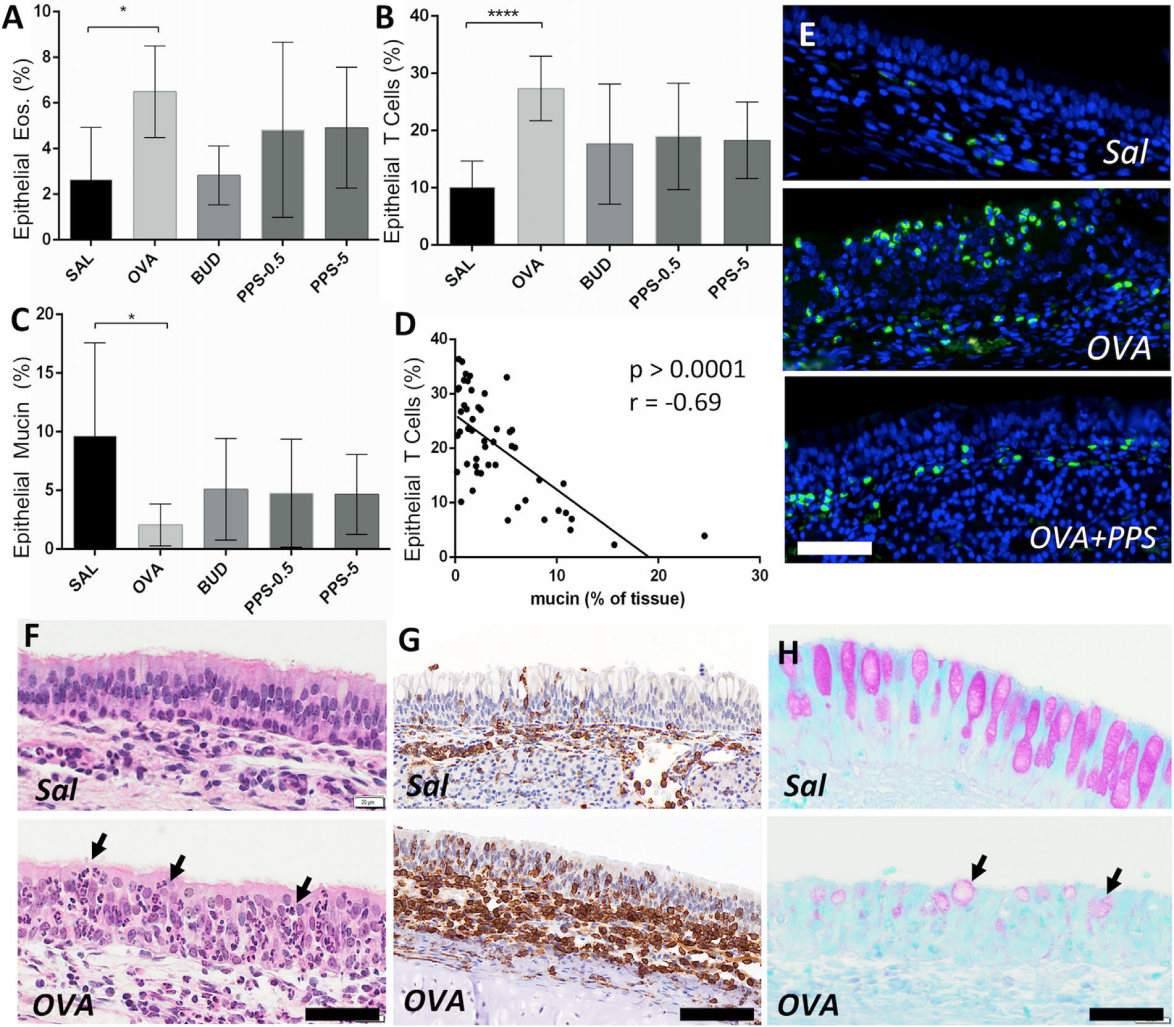
Impact of PPS and budesonide on histological nasal tissue parameters during the allergic late phase reaction. (A) Intraepithelial eosinophils (A), expressed as percent eosinophils per analysed tissue area and calculated by computerized image analysis of fluorochrome‐stained eosinophils (exemplified in E). Corresponding data for epithelial nasal tissue T lymphocytes are shown in B and G. Epithelial loss of PAS % mucin content is shown in C and H. Panel D shows a *Pearson* correlation analysis between epithelial T lymphocytes and epithelial loss of PAS positivity (i.e., mucus degranulation). (F) Hematoxylin stained nasal mucosa to exemplify the overall increased leukocyte tissue infiltration in allergen‐challenged animals (infiltrating leukocytes are exemplified by arrows). PPS‐0.5 and 5 correspond to a prophylactic topical dose of 0.5 and 5 mg/kg, respectively. OVA, ovalbumin; Sal, saline control. Bar codes in E–G = 70 µm, H = 50 µm.

Numbers of tissue T cells in the nasal airway epithelium were evaluated as percentage of tissue positive for the pan T lymphocyte marker CT5 (Fig. 5 B,G). Actively sensitized and allergen‐challenged animals displayed significantly increased T cell immunoreactivity compared to non‐allergic controls (*P* < 0.001, Fig. 5B). A clear trend toward a reduction of T cells were observed in both the Budesonide and PPS‐treated groups, although the effects did not reach statistical significance compared to sham‐treated OVA‐challenged animals (Fig. 5B).

Epithelial eosinophil numbers demonstrated significant differences between the saline and allergen‐challenged OVA groups (Fig. 5A). In similarity to T lymphocytes, eosinophil numbers displayed clear, albeit not statistically secured, reduction after treatment with Budesonide or PPS (Fig. 5A,E).

Epithelial PAS staining of goblet cell mucins was quantified in order to evaluate the reduction in epithelial PAS content occurring after allergen induced hyper secretion 33. As expected, the level of epithelial PAS content was significantly reduced in actively challenged animals (Fig. 5C,H). There was a similar and non‐statistical trend toward normalization of this parameter with budesonide and PPS treatment (Fig. 5C).

On the whole, there was a strong correlation between loss of epithelial PAS positive mucus and intraepithelial T cells (Fig. 5D, *P* < 0.0001, *r* = 0.69) or between PAS reduction and eosinophils (*P* < 0.01, *r* = 0.36).

## Discussion

The present study reveals several novel aspects regarding the capacity of PPS to modify an allergic response. Although it is evident that PPS may bind several different types of target molecules, the present study suggests that a key feature of any such anti‐allergic action is likely to involve binding and neutralization of Th2 cytokines. For example, using surface plasmon resonance technology, this study is the first to report that PPS binds with strong affinity to the Th2 cytokines IL‐4, IL‐5, and IL‐13. Indeed, the binding affinity was stronger than for the established Th2 cytokine binding of heparin. The demonstration of the competitive binding of PPS to IL‐4, IL‐5, and IL‐13 further suggests that PPS binds to the same structural regions that heparin binds. The structural interactions of heparin to the four α‐helical bundle cytokine family (IL‐4, IL‐5, and IL‐13) have been predicted by molecular modeling by Mulloy and Forster 23 and substantiated by biological data 22.

Importantly, that the binding of PPS to Th2 cytokines translates into biological response modification was here demonstrated by the present inhibition of IL‐4‐, IL‐5‐, and IL‐13‐dependent cellular proliferation of target cell‐lines. This is consistent with PPS binding to the region of the cytokine that interacts with the cytokine‐receptor thus resulting in the blockade of functional signal transduction. Hence, it can be concluded that PPS is a Th2 cytokine antagonist and although other types of responder cells are targeted in allergic disease, it is likely to have a broad inhibitory effect on Th2‐mediated immunological responses.

It is noteworthy that removal of anticoagulant oligosaccharides from low molecular weight heparins such as Enoxaparin to enrich for Th2 cytokine antagonistic activity are being attempted despite long standing technical problems 34. Thus, PPS having the advantage of being a weak anticoagulant (1/15 of heparin) could be repurposed as an anti‐inflammatory and ‐allergic treatment for AR without the need to modify the active pharmaceutical agent. Corroboration of the in vitro findings that PPS is a Th2 cytokine antagonist was in this study suggested by in vivo attenuation of the late phase Th2‐driven eosinophilic infiltration in the nasal lumen in an ovalbumin‐induced translational model of AR in guinea pigs 30, 31, 35. Previous studies have identified elevated Th2 cytokines in the present type of GP rhinitis model 36, 37 and that development of the allergen‐induced eosinophilia is IL‐5 dependent and can be blocked by the IL‐5 neutralizing antibody TRFK‐5 37.

In the present study, PPS significantly reduced the influx of multiple leukocytes, including eosinophils, into the nasal lumen. A similar clear, but not statistically secured, effect was also found for eosinophils and T‐lymphocytes in the nasal mucosal tissue. If PPS exerts its in viv*o* neutralizing effect differentially at lumen versus tissue sites remains to be explored.

PPS was in this study administered topically via the intra‐nasal route. A potential weakness is that the nasal bio‐distribution was, however, not investigated in this study. Nevertheless, potential sites of PPS action are worthy of consideration. Although tissue penetration of PPS with a large MW (4–6 kDa) may be impeded in healthy individuals 38, studies by Andersson et al. 39 show that treatment of the nasal cavity with detergent enabled the tracer molecule, PS15000 of MW 14.7 kDa to permeate across the nasal mucosa of healthy subjects. There is accumulating evidence in human AR subjects and in animal models that IL‐4 has a role in nasal barrier dysfunction associated with changes in intercellular tight junction protein expression 40 and the potassium channel protein, TREK‐1 41. Moreover, the dysfunctional epithelial barrier in AR in clinical and experimental scenarios has been suggested to permit large molecules (e.g., FITC‐dextran MW of 4 kDa) to permeate into the sub‐mucosa 40, 41. Based on this evidence, it is plausible that PPS may permeate across the disrupted epithelial layer in AR and thus enter the submucosal layer where it can interact with extracellular cytokines.

Contemporaneously, PPS may localize directly onto the nasal epithelial cells and function to protect the epithelial barrier. Confirmation of preferential localization of PPS to the surface epithelium is provided by the autoradiographic detection of tritium‐labeled PPS in the urinary tract in rats administered either orally or intravesically 42. For the clinical treatment of interstitial cystitis with PPS, it has been proposed that PPS acts by replacing the damaged GAG layer that lines the bladder 43 and that this effect was confirmed by reductions in bladder permeability based on the potassium sensitivity test 44.

The present demonstration of a significant inhibitory effect by PPS on extravasated plasma proteins is an important feature of PPS as a potential anti‐allergic drug. Plasma extravasation is a physiological defense response that also is a pathophysiological hallmark of AR 45. During this process, endothelial gap formation creates a flow of non‐sieved bulk plasma into the tissue and the airway lumen 46. The biological purpose is to “kick start” the inflammation and pave the way for the subsequent build‐up of an immunological and cellular immune response. The present model, which mimics the biphasic extravasation response typically seen in allergic patients 47, was used to measure extravasation during the allergic late phase reaction. Hence, it is likely that also for this parameter the inhibitory effect by PPS may be a result of Th2 neutralization. However, a mast cell stabilizing effect is also possible since PPS has been shown to have a mast cell stabilizing effect 21 and histamine is a major inducer of plasma extravasation. Indeed, the contribution of mast cell stabilization merits further investigation since an acute anaphylactic mast cell degranulation likely facilitates the subsequent late phase reaction. As a side note, during the initiation of this study, it was noted that some animals died accidentally from acute anaphylaxis after accidental inspiration of allergen to the bronchi and bronchioles. Interestingly, PPS‐treated animals had virtually no such anaphylactic reactions. Whether or not PPS and its known MC stabilizing effects protect from acute anaphylaxis and bronchospasm remains highly speculative and the subject deserves further investigation. Whereas the previous careful validation of the present late phase model should be considered a significant strength of the present in vivo part, it should be noted that it also remains to be established to what extent PPS counteracts a chronic allergic inflammation or other long‐term effects such as airway remodeling.

In our in vivo study, the clinical formulation of the corticosteroid budesonide (Rhinocort® Aqua) was used as reference drug. Across the explored parameters, PPS and budesonide displayed similar anti‐inflammatory profiles. Despite the multiple targets that PPS binds the therapeutic consequences are likely immunomodulation rather than immunosuppression like for corticosteroids. The long‐standing clinical use of PPS as an oral formulation as well as an injectable without reports of immunosuppressive side effects such as infection attest to the safety of PPS as an anti‐inflammatory agent 48. That this safety aspect is valid for also nasal topical delivery is also suggested by recent toxicology studies in rats where a twice a day high‐dose nasal administration of PPS for 28 days was well tolerated (Charles River testing facility study 20085506).

In conclusion, this study highlights out PPS as a potent Th2 cytokine‐binding molecule that has broad anti‐inflammatory effects in a validated animal model of AR. As such PPS fulfills the role as a potential first‐in class agent for the treatment of AR having a distinct mechanism of action to steroids but with broad Th2 antagonistic and mast cell stabilizing effects. Since preliminary phase‐1 clinical data indicate that topical PPS nasal spray is well tolerated also in human subjects further studies exploring the clinical efficacy of PPS in rhinitis patients are now highly warranted.

## Authors’ Contributions

C.S. has been involved in in vivo histology and immune cell analysis. M.M. has been involved in data interpretation and manuscript writing. P.J. and J.J performed histological analyses. R.K. has contributed to the background research and feedback on the manuscript. X.W. contributed to design of the in vivo study and interpretation of BAL results. J.E. contributed to the study design, overall study layout, and manuscript writing.

## Conflicts of Interest

R.K. is an employee of Paradigm Biopharmaceuticals, J.E. is advisor to Paradigm Biopharmaceuticals and founder of the CRO Company Medetect AB. All other authors declare no competing financial interests. All animal experiments in the present study have been approved by the local Malmo/Lund Ethical Committee (DNo: M7907).
